# Flavonoids as Promising Akt1 Inhibitors in Cancer Medicine: Insights From Molecular Docking, Dynamics, DFT Calculations, and In Vitro Validation

**DOI:** 10.1002/cnr2.70315

**Published:** 2025-08-12

**Authors:** Shokoofeh Jamshidi, Ali Eghbalian, Setareh Shojaei, Amir Taherkhani, Mehran Feizi‐Dehnayebi

**Affiliations:** ^1^ Department of Oral and Maxillofacial Pathology, School of Dentistry Hamadan University of Medical Sciences Hamadan Iran; ^2^ Research Center for Molecular Medicine, Institute of Cancer, Avicenna Health Research Institute Hamadan University of Medical Sciences Hamadan Iran; ^3^ Department of Organic Chemistry, Faculty of Chemistry Alzahra University Tehran Iran

**Keywords:** Akt1, cancer, drug, flavonoid, molecular docking

## Abstract

**Background:**

The PI3K/Akt/mTOR signaling pathway is commonly deregulated in different types of cancers, contributing to tumor proliferation, persistence, and resistance to treatment. Akt1, a crucial kinase within this pathway, plays a critical role in tumor progression and the occurrence of therapeutic resistance. The emergence of resistance is a significant challenge in cancer therapy. Targeted therapies offer a promising method to overcome this challenge. Akt1 presents a promising target for therapeutic intervention.

**Aims:**

This study aimed to evaluate the binding affinities of 61 flavonoid‐derived natural compounds to the Akt1 ATP‐binding site using molecular docking with AutoDock to identify potential Akt1 inhibitors.

**Methods:**

Cross‐validation and Density Functional Theory analysis were conducted utilizing the SwissDock server and the Gaussian 09 W software suite for the top‐ranked compounds. Following energy minimization, semi‐flexible docking of flavonoids and the control inhibitor Ipatasertib was performed against the Akt1 ATP‐binding pocket. Binding modes were analyzed using Discovery Studio Visualizer. Molecular dynamics simulations were conducted to assess the conformational stability and binding durability of the highest‐scoring Akt1 inhibitor complex identified through molecular docking analyses. The pharmacokinetics and toxicity properties of the most potent Akt1 inhibitors were evaluated using the PreADMET tool. Also, the effect of the most potent Akt1 inhibitor on cell viability was studied in vitro through the 2,5‐diphenyl‐2H‐tetrazolium bromide approach. Besides, the most promising compound was evaluated for its impact against the FOXO3 (an Akt1 downstream target) gene expression in MCF‐7 cells.

**Results:**

Kaempferol 3‐rutinoside‐4′‐glucoside and Kaempferol 3‐rutinoside‐7‐sophoroside displayed exceptional binding affinities (Δ*G*_binding_ = −21.79 and −20.73 kcal/mol; Ki = 106.03 aM and 640.24 aM), surpassing Ipatasertib (Δ*G*_binding_ = −9.98 kcal/mol; Ki = 48.29 nM). Kaempferol 3‐rutinoside‐4′‐glucoside achieved a stable binding conformation within the Akt1 catalytic domain after 30 ns of molecular dynamics simulation. The compound Kaempferol 3‐rutinoside‐4′‐glucoside was observed to suppress cell proliferation in MCF‐7 cell lines. This effect was accompanied by an upregulation of FOXO3 expression, suggesting a connection to the induction of the apoptosis pathway.

**Conclusions:**

Computational analyses identified flavonoids, particularly Kaempferol glycosides, as potential Akt1 inhibitors with significantly higher predicted binding affinities than Ipatasertib. These findings warrant further exploration of the therapeutic potential of flavonoids for cancers driven by Akt1 hyperactivation.

## 1 Introduction

The PI3K/Akt/mTOR signaling pathway is frequently dysregulated in various types of cancers. Aberrant activation of this pathway promotes tumor formation through diverse mechanisms, including enhanced cell proliferation, survival, invasion, and angiogenesis [1, 2, 3]. Akt1, a key serine/threonine kinase within this pathway, plays a critical role in tumor progression and the development of therapeutic resistance. It has been implicated in the pathogenesis of numerous malignancies, including breast, ovarian, lung, and pancreatic cancers [4, 5, 6, 7]. Akt1 enhances cell proliferation by modulating cell cycle regulators such as cyclin D1, p21, and p27 and suppressing apoptosis (p53 pathways), consistent across breast, cervical, and other cancers [8, 9]. Its role in metabolic reprogramming, particularly via phosphorylation‐induced cytoplasmic retention of ME2 and assembly of glycolytic enzyme complexes, represents a novel and critical mechanism by which Akt1 supports the bioenergetic demands of tumor cells [10]. This metabolic switch aligns with the Warburg effect, a hallmark of cancer metabolism, and underscores Akt's capacity to coordinate growth signals with metabolic adaptation. In addition, Akt1 functions downstream of PI3K and PDK1. Activated by these kinases or growth factors like IGF‐IR, Akt1 phosphorylates many substrates, including critical components of mTORC1, such as S6K1 and 4E‐BP1 [11]. This phosphorylation cascade profoundly influences cellular processes such as protein synthesis, glucose metabolism, and cell cycle progression [12]. Notably, hyperactivation of Akt1 has been observed in approximately half of breast cancers [13], often attributed to mutations or amplifications within the PI3K/Akt/mTOR axis. Furthermore, the emergence of resistance is a significant challenge in cancer therapy.

Combination therapies offer a promising strategy to overcome this hurdle. One approach involves combining Akt inhibitors with subtypes like ATP‐competitive (e.g., Ipatasertib), allosteric (e.g., MK‐2206), and lipid‐based targeting pleckstrin homology domains [14] alongside mTOR inhibitors like everolimus or temsirolimus. This synergistic strategy targets multiple nodes within the PI3K/Akt/mTOR pathway, leading to improved patient outcomes. Additionally, preclinical studies suggest that combining Akt inhibitors with conventional chemotherapies or targeted agents like PARP inhibitors yields synergistic effects [2, 15, 16]. Furthermore, research by Simpson et al. [17] underscores the therapeutic potential of targeting the PI3K/Akt/mTOR pathway in head and neck squamous cell carcinoma. Their work highlights how combining inhibitors targeting downstream effectors like Akt can potentially overcome resistance to cetuximab.

The exploration of natural products as potential therapeutics has gained significant momentum. A vast array of bioactive compounds from natural sources is under preclinical and clinical investigation for their ability to treat various diseases [18]. Notably, flavonoids, a diverse class of polyphenols found in plants, vegetables, and fruits, have demonstrated beneficial properties in cancer medicine [19]. They have garnered considerable interest due to their potential to inhibit the PI3K/Akt/mTOR pathway. Studies have shown that Luteolin, Myricetin, and Naringenin can suppress cancer cell survival and proliferation through this pathway [20, 21, 22, 23]. Luteolin, for example, has been demonstrated to induce apoptosis by inhibiting Akt phosphorylation and mTOR activity [21]. Similarly, naringenin can downregulate Akt phosphorylation, leading to apoptosis and cell cycle arrest in cancer cells [23]. As well, Baicalin, another natural product, exhibits promise in treating fibrotic lung diseases by targeting the PI3K/Akt pathway. Further studies in animal models also suggest that Baicalin treatment reduces fibroblast proliferation and fibrotic changes [24]. These findings highlight the potential of natural flavonoids to act as therapeutic agents by modulating the PI3K/Akt/mTOR pathway.

Molecular docking studies, as a scientific branch in bioinformatics, have become a cornerstone of drug discovery. This computational technique empowers researchers to predict interactions between small molecules and their biological targets. In the context of our current study, molecular docking provided distinct advantages over traditional experimental screening methods by enabling systematic evaluation of large compound libraries against specific protein targets without the resource‐intensive synthesis and biological testing required in conventional approaches. This computational methodology builds explicitly upon previous research by incorporating advanced scoring functions and ensemble docking protocols, thereby addressing the limited accuracy and flexibility constraints that have hampered earlier computational studies. Molecular docking has significantly streamlined drug discovery by enabling high‐throughput virtual screening and rational drug design, reducing time and cost. Its ability to predict binding affinities and modes proves particularly valuable, as this information is crucial for identifying promising lead compounds. The computational framework implemented in this study enhanced predictive reliability by integrating multiple conformational states and dynamic binding site analysis, capabilities that experimental methods cannot achieve with comparable efficiency and throughput. Furthermore, molecular docking extends its utility by effectively predicting drug‐excipient interactions, enzyme‐peptide interactions, and the stability of drug inclusion complexes. These predictions offer invaluable insights for enhancing protein stability, solubility, dissolution rates, and a drug's bioavailability [25]. Besides, the recent Food and Drug Administration legislation eliminates the mandatory use of animal testing for drug approval. This aligns with animal welfare advocates and encourages alternative methods like computer modeling and organ‐on‐a‐chip technology, which have gained traction in recent years [26, 27]. This shift signifies growing confidence in the reliability of computational approaches for drug discovery, particularly methodologies like ours that demonstrate enhanced predictive accuracy and broader analytical scope compared to earlier computational models.

Due to the significant role of Akt1 in tumor progression and drug resistance, coupled with the documented efficacy of flavonoids in targeting the PI3K/Akt/mTOR pathway, the present study aimed to evaluate the binding affinity of a set of 61 herbal flavonoids to the Akt1 ATP‐binding domain. This was done by using molecular docking analysis, followed by molecular dynamics (MD) simulations and Density Functional Theory (DFT) analysis. Subsequently, the impact of the most potent flavonoid on cell viability was evaluated in vitro through the 2,5‐diphenyl‐2H‐tetrazolium bromide (MTT) assay. Further, the top‐ranked flavonoid was assessed for its effect against the FOXO3 (an Akt1 downstream target) gene expression in Human MCF‐7 cancer cells.

## 2 Methods

### 2.1 Pre‐Docking Preparations

In this investigation, the Protein Data Bank (PDB), available at https://www.rcsb.org/ [28], was accessed to retrieve the crystallographic structural data of the Akt1 protein (PDB ID: 4GV1, resolution: 1.49 Å). This structure, comprising a single polypeptide chain with 340 amino acid residues, underwent energy minimization using the Swiss‐PdbViewer version 4.1.0 software.

A comprehensive evaluation of the 4GV1 3D structure was conducted using the PROCHECK server (https://saves.mbi.ucla.edu/) [29]. This server illustrates the phi (ϕ) and psi (ψ) dihedral angle distribution for the protein's residues.

Using BIOVIA Discovery Studio Visualizer (DSV) software, available at https://www.3ds.com/products/biovia/discovery‐studio/visualization, the interactions between Capivasertib (PubChem ID: 25227436), an internal inhibitor in the 4GV1 structure, and the active site residues were analyzed. Critical residues for ligand binding, including Leu156, Gly157, Gly162, Val164, Ala177, Met227, Ala230, Glu234, Glu278, Asn279, and Met281, were identified. Consequently, a grid box with dimensions of 60 × 60 × 60 and a center at *X* = −20.556, *Y* = 5.617, and *Z* = 11.523 was established around the Akt1 catalytic pocket to define favorable ligand interactions precisely.

A set of 61 flavonoid compounds was selected for docking analysis. Their binding affinities towards the Akt1 ATP‐binding cleft were evaluated through molecular docking simulations and compared to the established inhibitor, Ipatasertib, which served as a standard drug. Following established protocols, all small molecules underwent energy minimization using the HyperChem software, available at http://hypercubeusa.com. Polar hydrogens were added to prepare the 4GV1 structure for docking simulations with AutoDock 4.0, available at https://ccsb.scripps.edu/mgltools/, and Kollmann charges were assigned to the structure. The protein structure was further processed to incorporate rotational freedom for the small molecules and account for their local charges. The maximum number of evals, rate of gene mutation, rate of crossover, and variance of the Cauchy distribution for gene mutation were set to 2 500 000, 0.02, 0.8, and 1.0, respectively. Finally, the Protein Data Bank with partial Charge and atom Type (PDBQT) was employed to generate the necessary input files for docking simulations, executed through the Cygwin64 Terminal interface (https://www.cygwin.com) [30].

### 2.2 Molecular Docking and Structural Analyses

The Cygwin tool was used for calculating Δ*G*_binding_ and Ki values, as well as details of binding energies between the studied ligands and the Akt1 active site. This software retrieves its input data from the AutoDock software. The docking methodology was semi‐flexible, allowing for conformational flexibility within the protein during the simulation process. AutoDock combines its primary docking algorithm, the Lamarckian Genetic Algorithm, with a semi‐empirical free energy force field, which functions as its scoring mechanism [31].

This study evaluated the binding affinities of 61 flavonoid compounds and Ipatasertib towards the ATP‐binding site of Akt1, a protein of interest. A grid box with specific dimensions and spacing was defined around the Akt1 ATP‐binding site to pinpoint favorable ligand interactions accurately. To account for potential variations in the ligand's orientation, 50 conformations were generated for each compound. This was done using the Cygwin tool.

The analysis calculated the binding free energy for all compounds, indicating the strength of the ligand–protein interaction. From each set of conformations, the conformation with the most negative Δ*G*_binding_ value (representing the strongest predicted binding affinity) was selected from the most populated cluster (the group comprising the most similar conformations). Subsequently, DSV aided in analyzing the interaction modes between the selected ligand conformations and the Akt1 protein, providing a three‐dimensional representation of potential binding poses.

### 2.3 Cross‐Validation Study

A multi‐tiered validation strategy was implemented to enhance the robustness and reliability of docking predictions. Initial docking studies used AutoDock 4.0, a common software with a semi‐flexible protocol that allows ligand flexibility while keeping the protein rigid. Though AutoDock 4.0 permits some protein flexibility by designating specific side chains as flexible [32], it primarily focuses on ligand conformational sampling.

To further validate these results, independent docking simulations were run on the SwissDock server (https://www.swissdock.ch/) using two algorithms:

1. Docking with attractive cavities, which performs blind docking across the entire protein surface. This eliminates bias from predefined grid boxes and enables unbiased exploration of potential binding sites [33, 34].
2. Docking with AutoDock Vina, which performs docking with a specified binding site. The box center was positioned at coordinates (−20.556, 5.617, 11.523) along the *X*, *Y*, and *Z* axes, respectively. The box dimensions were uniformly set to 20 units for all axes.

By combining different docking platforms with varying receptor flexibility and search algorithms, this multi‐tiered approach provided a robust assessment of predicted binding modes. The convergence of results from both AutoDock 4.0 and SwissDock, despite their methodological differences, significantly strengthens confidence in the identified binding interactions between the flavonoid compounds and the Akt1 ATP‐binding site.

### 2.4 Molecular Dynamics Simulations

The stability and dynamic action of Akt1 protein were evaluated through MD simulations, examining both the most promising inhibitor candidate identified from docking studies and the established drug Ipatasertib. The simulations were conducted using Discovery Studio Client (version 16.1.0.15350). The computational analysis was performed on a high‐performance system featuring a 24‐core Intel i9‐13900KF processor and 64GB DDR5 RAM in a 64‐bit environment.

Moreover, the MD simulations were performed using the CHARMM force field, with partial atomic charges derived directly from its parameters. To prepare the system, the protein‐ligand complex was solvated using the TIP3P water model in a rectangular periodic box. A crucial step was maintaining a minimum distance of 10 Å between the protein and the box boundaries. The complete simulation system for free Akt1 included 37 421 atoms: 5358 from the protein, 32 001 from water, and 34 Na^+^with 28 Cl^−^ ions, which were added to ensure electroneutrality and a physiological ionic strength of 0.145 M NaCl. It is worth noting that Kaempferol 3‐rutinoside‐4′‐glucoside comprised 107 atoms, while Ipatasertib had 64 atoms. The solvated system then underwent an energy minimization step, followed by equilibration phases. Finally, production MD simulations were carried out for 110 ns. Throughout these simulations, a Nosé‐Hoover thermostat maintained the temperature at 310 K, and a Parrinello‐Rahman barostat kept the pressure at 1 atm. Integration was performed using the leap‐frog algorithm with a 2 fs time step, and the Particle Mesh Ewald (PME) method was employed for calculating long‐range electrostatic interactions.

Throughout the simulations, protein stability was monitored through root mean square deviation (RMSD) calculations of the Akt1 backbone atoms. Additionally, root mean square fluctuation (RMSF) analysis was employed to assess the temporal displacement of protein residues relative to their reference positions, providing insights into local structural flexibility. Furthermore, the solvent‐accessible surface area (SASA) and radius of gyration (ROG) of Akt1 were calculated.

### 2.5 Pharmacokinetic and Toxicology Prediction

The PreADMET server (https://preadmet.webservice.bmdrc.org/) was used to evaluate ADMET properties (Absorption, Distribution, Metabolism, Excretion, and Toxicity) of the top‐ranked flavonoids, including Kaempferol 3‐rutinoside‐4′‐glucoside and Kaempferol 3‐rutinoside‐7‐sophoroside. PreADMET is a web‐based in silico application for predicting ADMET data and constructing drug‐like libraries [35, 36].

### 2.6 Density Functional Theory (DFT) Calculation

Structural optimization of the top‐three ranked compounds was performed using Density Functional Theory (DFT) calculations [37]. The Gaussian 09 W software suite, available at https://gaussian.com/ [38], was employed with GaussView 6.0 for visualization. The B3LYP functional and the 6‐311G(d,p) basis set were utilized for carbon, hydrogen, and oxygen atoms, respectively. The optimized molecular geometries obtained from these calculations were the foundation for subsequent investigations. These investigations included the analysis of molecular electrostatic potential (MEP) maps and the examination of frontier molecular orbitals (HOMO and LUMO). These analyses provided valuable insights into the potential reactive sites, biological activity, and overall stability of the molecules.

### 2.7 Cell Viability Assay

This study evaluated the cytotoxicity of the top‐ranked inhibitor through a tetrazolium 3‐(4,5‐dimethylthiazol‐2‐yl)‐2,5‐diphenyltetrazolium bromide (MTT) assay (Sigma, USA). Initially, 1 × 10^4^ MCF‐7 cells were seeded into each well of 96‐well tissue culture plates and allowed to grow overnight. Various concentrations of the most effective Akt1 inhibitor were then prepared in a 10% FBS‐high glucose DMEM medium and introduced to the cells.

Following a 24‐h incubation period at 37°C, 10 μL of MTT dye (5 mg/mL) was added to each well. The plates were then incubated for another 4 h at 37°C with 5% CO_2_. During this time, metabolically active, viable cells transformed the MTT into a purple formazan product. The medium was subsequently removed, and the insoluble formazan precipitate was dissolved using DMSO.

An ELISA microplate reader was used to measure the absorbance at 570 nm, with a reference wavelength of 630 nm. This measurement enabled the calculation of cell viability percentages. The resulting data underwent analysis using both Excel and GraphPad Prism 5 software.

### 2.8 RNA Isolation and Quantitative Reverse‐Transcription Polymerase Chain Reaction (RT‐qPCR)

After 24 h of treatment with Kaempferol 3‐rutinoside‐4′‐glucoside total RNA was extracted from MCF‐7 cells using an RNX‐Plus Kit (Cinnagen, Tehran, Iran). RNA quantity and quality were assessed with a NanoDrop. Subsequently, 2 μg of total RNA was reverse‐transcribed into complementary DNA (cDNA) using a first‐strand cDNA synthesis kit (Cinnagen, Tehran, Iran).

Real‐time PCR was then performed with SYBR green master mix (Ampliqon, Denmark) and specific primers for FOXO3 (sequences in Table 1). Thermal cycling involved an initial denaturation at 95°C for 15 min, followed by 40 cycles of denaturation at 95°C for 15 s, annealing at 58°C (FOXO3) or 53°C (GAPDH) for 30 s, and extension at 72°C for 30 s. A Roche 96 Light Cycler instrument (Germany) was used for this assay. Gene expression levels were normalized against GAPDH (internal control), and the relative expression of FOXO3 was quantified using the 2^−ΔΔCT^ method.

**TABLE 1:** Specific primer sequences for qRT‐PCR.

Gene	Forward strand	Reverse strand	References
FOXO3	5´‐TCTACGAGTGGATGGTGCGTT‐3′	5′‐CGACTATGCAGTGACAGGTTGT‐3′	Albamonte et al. [39]
GAPDH	5´‐CCCGTAGACAAAATGGTGAAGGTC‐3′	5′‐GCCAAAGTTGTCATGGATGACC‐3′	Liu et al. [40]

Abbreviation: qRT‐PCR, quantitative reverse transcription PCR.

## 3 Results

### 3.1 Binding Affinities

Akt1 is a critical node in cancer progression, influencing diverse signaling pathways. Its role has been implicated in a multitude of malignancies, including lung, breast, and esophageal squamous cell carcinoma, where it promotes tumor initiation, progression, and metastasis [41, 42, 43, 44, 45]. This underscores the promise of targeting Akt1 with specific therapies. Notably, Akt1 activity is linked to aggressive features and metastatic potential in these cancers. Therefore, targeting Akt1 or its regulators holds promise as a novel therapeutic strategy against various cancers.

**FIGURE 1:**
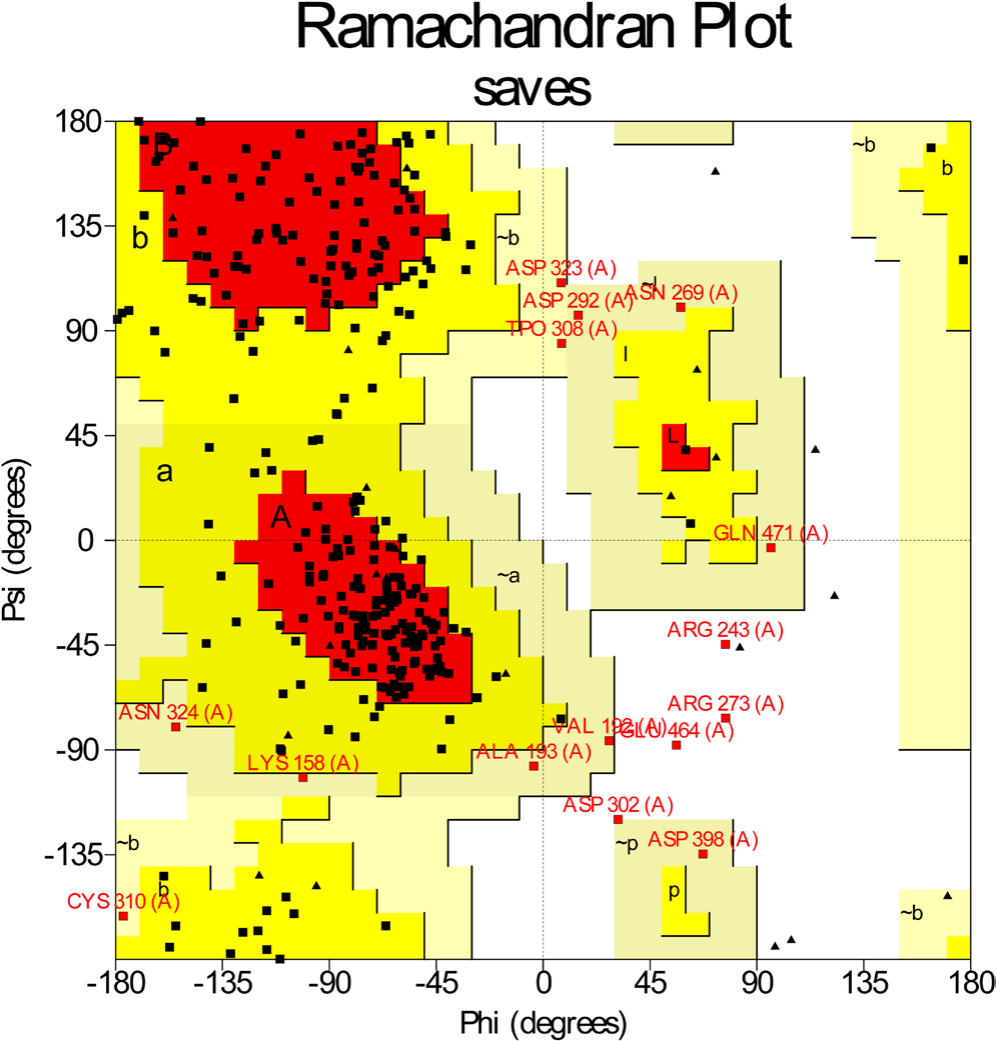
Ramachandran plot illustrating the quality of the 4GV1 three‐dimensional model. This plot reveals the distribution of residues, with 65.5% located in favored regions, 29.5% in additional allowed regions, 3.8% in generously allowed regions, and only 1.4% in disallowed areas.

The Ramachandran plot from the PROCHECK server revealed that 65.4% of residues were in the most favorable regions, 29.5% in supplementary allowed, 3.8% in generously allowed, and 1.4% in disallowed areas (Figure 1).

According to the present study, Kaempferol 3‐rutinoside‐4′‐glucoside and Kaempferol 3‐rutinoside‐7‐sophoroside exhibited the most potent inhibitory effects on the Akt1 ATP‐binding domain among the 61 flavonoids. Notably, these compounds were predicted to restrict enzyme activity in the atto molar range, demonstrating exceptional efficacy. The Δ*G*_binding_ scores for these phenolics, which reflect binding affinity, were remarkably high at −21.79 and −20.73 kcal/mol, respectively. Sophoraflavanone G, Amentoflavone, Orientin, Isoquercitrin, Quercetin‐3‐rhamnoside, Nicotiflorin, and Rutin also emerged as promising Akt1 inhibitors, displaying Ki values in the picomolar range. Consequently, these nine plant‐derived phenolics were selected for further analysis following the docking simulations.

**FIGURE 2:**
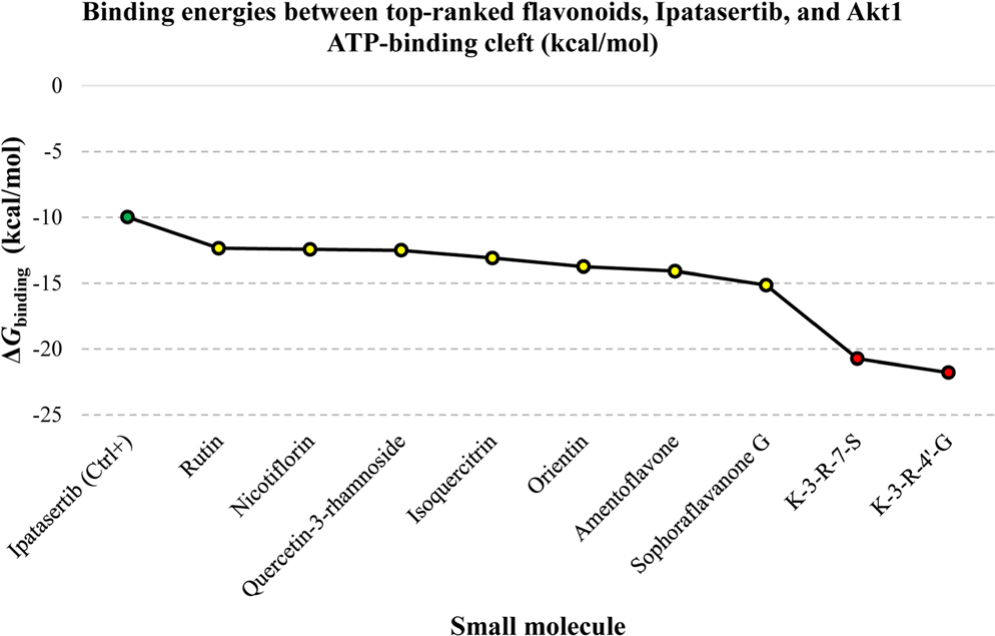
Δ*G*_binding_ values between top‐ranked flavonoids, a reference drug, and the Akt1 active site. The X‐axis demonstrates the names of compounds, and the Y‐axis illustrates their Gibbs free binding energy in kcal/mol units. The green diamond demonstrates the control inhibitor, the red spots represent the most potent Akt1 inhibitors in this study with *K*i values at the attomolar scale, and the yellow spots indicate herbal isolates with *K*i values at the picomolar scale. Akt1, RAC‐alpha serine/threonine‐protein kinase; *K*i, inhibition constant.

For comparison, the Δ*G*_binding_ and Ki values predicted for the control inhibitor, Ipatasertib, were −9.98 kcal/mol and 48.29 nM, respectively. This suggests that the top nine flavonoids exhibited a superior binding affinity for the Akt1 ATP‐binding site compared to the reference drug. Table 2 summarizes the Δ*G*_binding_ scores and Ki values for all tested flavonoids alongside Ipatasertib. To gain deeper insights into the binding interactions, Table 3 presents a detailed breakdown of the binding energies (including intermolecular energy, total internal energy, torsional free energy, and unbound systems energy) for the top‐ranked compounds and the reference drug. Finally, Figure 2 visually compares the Δ*G*_binding_ value of Ipatasertib with the strongest binding affinities observed for the identified flavonoids.

**TABLE 2:** Computational docking studies quantified binding energies and inhibition constants for interactions between the Akt1 catalytic domain and a panel of 61 flavonoids, compared with Ipatasertib as the reference inhibitory compound.

Ligand name	PubChem ID	Binding energy (kcal/mol)	Ki	Ki (based on nM)
Kaempferol 3‐rutinoside‐4′‐glucoside	44 258 844	−21.79	106.03 aM	1.0603e‐7
Kaempferol 3‐rutinoside‐7‐sophoroside	44 258 853	−20.73	640.24 aM	6.4024e‐7
Sophoraflavanone G	72 936	−15.14	8.03 pM	0.00803
Amentoflavone	5 281 600	−14.09	46.93 pM	0.04693
Orientin	5 281 675	−13.75	83.93 pM	0.08393
Isoquercitrin	5 280 804	−13.10	248.60 pM	0.24860
Quercetin‐3‐rhamnoside	5 353 915	−12.51	680.10 pM	0.68010
Nicotiflorin	5 318 767	−12.44	757.94 pM	0.75794
Rutin	5 280 805	−12.35	880.03 pM	0.88003
Poncirin	442 456	−12.25	1.06 nM	1.06
Kaempferol 7‐O‐glucoside	10 095 180	−12.18	1.18 nM	1.18
Cynaroside	5 280 637	−11.96	1.71 nM	1.71
Astragalin	5 282 102	−11.87	1.98 nM	1.98
Procyanidin	107 876	−11.78	2.33 nM	2.33
Apigenin‐7‐glucoside	5 280 704	−11.76	2.41 nM	2.41
Vicenin‐2	442 664	−11.69	2.72 nM	2.72
Vitexin	5 280 441	−11.49	3.79 nM	3.79
Quercitrin	5 280 459	−11.46	3.95 nM	3.95
Eriocitrin	83 489	−11.45	4.05 nM	4.05
Quercetin	5 280 343	−10.80	12.20 nM	12.20
Herbacetin	5 280 544	−10.71	14.06 nM	14.06
Afzelin	5 316 673	−10.52	19.42 nM	19.42
Taxifolin	439 533	−9.99	47.37 nM	47.37
Tricetin	5 281 701	−9.55	100.21 nM	100.21
Diosmetin	5 281 612	−9.45	117.94 nM	117.94
Astragarin	9 911 508	−9.25	165.20 nM	165.20
Isorhamnetin	5 281 654	−9.14	200.66 nM	200.66
Dihydroquercetin	471	−9.07	224.18 nM	224.18
Xanthogalenol	14 309 735	−9.03	241.26 nM	241.26
Myricetin	5 281 672	−8.86	321.20 nM	321.20
Delphinidin	128 853	−8.80	356.29 nM	356.29
Fustin	5 317 435	−8.77	375.29 nM	375.29
Hemileiocarpin	629 440	−8.74	393.16 nM	393.16
Chrysin	5 281 607	−8.64	461.51 nM	461.51
Apigenin	5 280 443	−8.60	496.55 nM	496.55
Fisetin	5 281 614	−8.57	525.52 nM	525.52
Luteolin	5 280 445	−8.25	893.68 nM	893.68
Datiscetin	5 281 610	−8.23	930.86 nM	930.86
Catechin	9064	−8.22	946.74 nM	946.74
Pinostrobin	4 101 463	−8.15	1.05 uM	1050
XanthohuMol	639 665	−8.14	1.08 uM	1080
3‐O‐Methylquercetin	5 280 681	−8.13	1.10 uM	1100
Morin	5 281 670	−8.09	1.18 uM	1180
Licochalcone A	5 318 998	−8.03	1.31 uM	1310
Acacetin	5 280 442	−8.00	1.37 uM	1370
Epicatechin	1203	−7.97	1.44 uM	1440
Kaempferol	5 280 863	−7.96	1.47 uM	1470
Glabridin	124 052	−7.95	1.50 uM	1500
Isoliquiritigenin	638 278	−7.71	2.24 uM	2240
4′‐O‐methyl‐epicatechin	14 332 898	−7.68	2.34 uM	2340
Eriodictyol	440 735	−7.64	2.52 uM	2520
Epiafzelechin	443 639	−7.62	2.61 uM	2610
Hesperetin	72 281	−7.62	2.60 uM	2600
Ponciretin	25 201 019	−7.50	3.20 uM	3200
Cyanidin	128 861	−7.29	4.50 uM	4500
Laricitrin	5 282 154	−7.10	6.24 uM	6240
Formononetin	5 280 378	−6.98	7.68 uM	7680
Flavone	10 680	−6.93	8.37 uM	8370
Genkwanin	5 281 617	−6.89	8.84 uM	8840
Sinensetin	145 659	−6.43	19.34 uM	19 340
Nobiletin	72 344	−6.19	29.22 uM	29 220
Ipatasertib (Ctrl+)	24 788 740	−9.98	48.29 nM	48.29

Abbreviations: Akt1, RAC‐alpha serine/threonine‐protein kinase; Ctrl, control; *K*i, inhibition constant.

**TABLE 3:** Types of energies between the Akt1 enzyme, top‐ranked flavonoids, and Ipatasertib as the reference drug.

Ligand name	Intermolecular energy (kcal/mol)	Total internal energy (kcal/mol)	Torsional free energy (kcal/mol)	Unbound system's energy (kcal/mol)	Estimated free energy of binding (kcal/mol)
Kaempferol 3‐rutinoside‐4′‐glucoside	−16.36	−14.47	5.97	−3.07	−21.79
Kaempferol 3‐rutinoside‐7‐sophoroside	−15.98	−17.09	7.46	−4.89	−20.73
Sophoraflavanone G	−14.11	−4.35	2.68	−0.64	−15.14
Amentoflavone	−10.84	−7.45	3.28	−0.91	−14.09
Orientin	−7.02	−11.22	3.58	−0.92	−13.75
Isoquercitrin	−9.03	−10.2	3.88	−2.25	−13.1
Quercetin‐3‐rhamnoside	−9.27	−8.94	3.28	−2.42	−12.51
Nicotiflorin	−11.46	−8.61	4.77	−2.85	−12.44
Rutin	−11.71	−9.09	5.07	−3.38	−12.35
Ipatasertib (Ctrl+)	−10.61	−2.49	2.09	−1.03	−9.98

Abbreviations: Akt1, RAC‐alpha serine/threonine‐protein kinase; Ctrl, control.

### 3.2 Cross‐Validations

To further assess the binding affinities of the top ligands identified by AutoDock 4.0, the SwissDock server was employed to determine SwissParam and affinity scores. SwissParam estimates binding free energy, providing a valuable metric for comparing the relative binding strengths of different ligand‐receptor complexes [33, 46, 47, 48, 49].

Based on SwissParam scores, Kaempferol 3‐rutinoside‐4′‐glucoside and Kaempferol 3‐rutinoside‐7‐sophoroside showed the most favorable binding free energies, with Δ*G* values of −8.95 and −8.44 kcal/mol, respectively. These values significantly surpassed the reference drug's Δ*G* of 8.03 kcal/mol within the Akt1 active site.

Additionally, AutoDock Vina identified Amentoflavone as having the highest binding affinity to the Akt1 active site, followed by Ipatasertib, Nicotiflorin, Rutin, Orientin, and Kaempferol 3‐rutinoside‐4′‐glucoside (Table 4). Notably, AutoDock Vina could not process Kaempferol 3‐rutinoside‐7‐sophoroside due to its large molecular size.

**TABLE 4:** SwissDock tool calculated SwissParam and binding affinity scores between the Akt1 catalytic domain and top‐ranked flavonoids, compared with Ipatasertib as the reference inhibitory compound.

Ligand	SwissParam score (kcal/mol)	Calculated affinity using the AutoDock Vina (kcal/mol)
Kaempferol 3‐rutinoside‐4′‐glucoside	−8.95	−8.106
Kaempferol 3‐rutinoside‐7‐sophoroside	−8.44	NA
Sophoraflavanone G	−7	−7.433
Amentoflavone	−7.6	−9.892
Orientin	−8.05	−8.148
Isoquercitrin	−7.82	−7.843
Quercetin‐3‐rhamnoside	−7.59	−7.996
Nicotiflorin	−7.86	−9.003
Rutin	−7.68	−8.935
Ipatasertib (Ctrl+)	−8.03	−9.221

Abbreviations: Akt1, RAC‐alpha serine/threonine‐protein kinase; Ctrl, control; NA, not available.

The consistent high ranking of Kaempferol 3‐rutinoside‐4′‐glucoside across both AutoDock 4.0 and SwissDock analyses (using the attractive cavities algorithm) further supports its strong binding affinity within the Akt1 catalytic domain.

### 3.3 Stability Analysis

**FIGURE 3:**
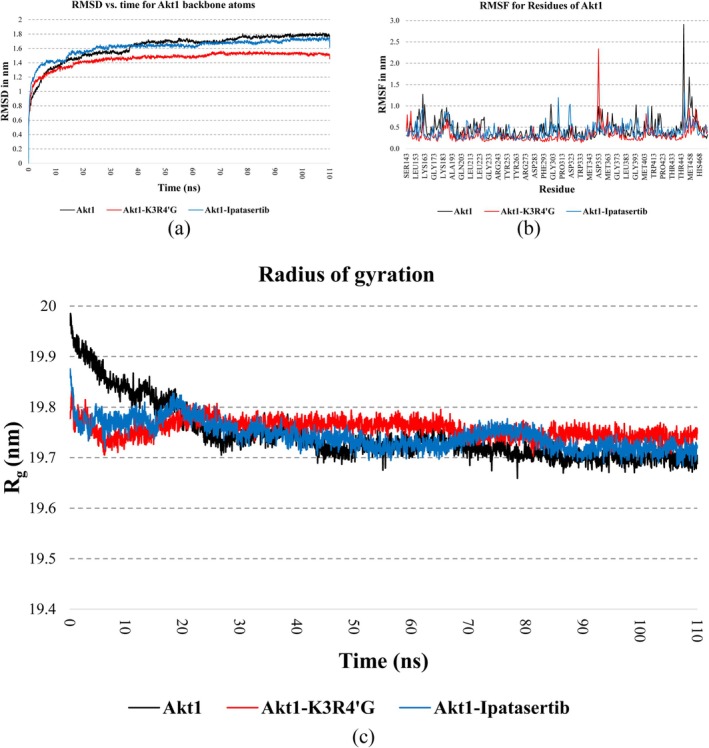
(a) RMSD, (b) RMSF, and (c) ROG for Akt1 backbone atoms in its free form and the presence of Kaempferol 3‐rutinoside‐4′‐glucoside and Ipatasertib during a 110 ns MD simulation. Akt1, RAC‐alpha serine/threonine‐protein kinase; RMSF, root mean square fluctuation; RMSD, root‐mean‐square deviation; ROG, radius of gyration.

Analysis of the RMSD trajectories demonstrated enhanced stability of Akt1 when bound to Kaempferol 3‐rutinoside‐4′‐glucoside surpassing the unbound receptor and the Ipatasertib‐bound complex in terms of conformational stability. Notably, the Akt1 backbone atoms achieved equilibrium approximately 30 ns into the simulation when Kaempferol 3‐rutinoside‐4′‐glucoside occupied the active site, as illustrated in Figure 3a. Comparative analysis revealed similar RMSD profiles for the unbound Akt1 and its complex with Ipatasertib; though the latter exhibited marginally improved backbone stability. These findings suggest that Kaempferol 3‐rutinoside‐4′‐glucoside may offer superior stabilization of the protein structure compared to the reference compound.

Examining RMSF profiles revealed notable differences in conformational mobility around the Akt1 active site region. The complex formed between Akt1 and Kaempferol 3‐rutinoside‐4′‐glucoside exhibited reduced local fluctuations in the Akt1 active site (Lys156, Gly157, Gly162, Val164, Met227, Ala230, and Glu234) compared to the unbound protein and the Ipatasertib‐bound complex, as depicted in Figure 3b. However, the Akt1 complexed with Kaempferol 3‐rutinoside‐4′‐glucoside shows high flexibility around some residues (353–363). These diminished fluctuation patterns strongly indicate that Kaempferol 3‐rutinoside‐4′‐glucoside effectively stabilizes the Akt1 active site region, potentially contributing to its enhanced binding characteristics.

It was also found that the ROG values were decreased for free Akt1 and Akt1 complexed with Ipatasertib and Kaempferol 3‐rutinoside‐4′‐glucoside during the 110 ns computer simulation. During the first 20 ns computer simulation, the ROG values were lower for the Akt1 complexed with Ipatasertib and Kaempferol 3‐rutinoside‐4′‐glucoside than for the free receptor (Figure 3c).

Further analysis indicated that the SASA for free Akt1 was lower than that of Akt1 complexed with Ipatasertib and Kaempferol 3‐rutinoside‐4′‐glucoside after MD simulations. Also, MD simulation reduced the SASA for free Akt1 and Akt1 complexed with Ipatasertib and Kaempferol 3‐rutinoside‐4′‐glucoside (Table 5).

**TABLE 5:** The solvent‐accessible surface area in square Angstroms (Å^2^) units for Akt1.

Situation	Akt1	Akt1‐Ipatasertib	Akt1‐Kaempferol 3‐rutinoside‐4′‐glucoside
Before MD	15 560	15280.8	15240.5
After MD	14427.7	14605.7	15152.7

Abbreviations: Akt1, RAC‐alpha serine/threonine‐protein kinase; MD, molecular dynamics.

**FIGURE 4:**
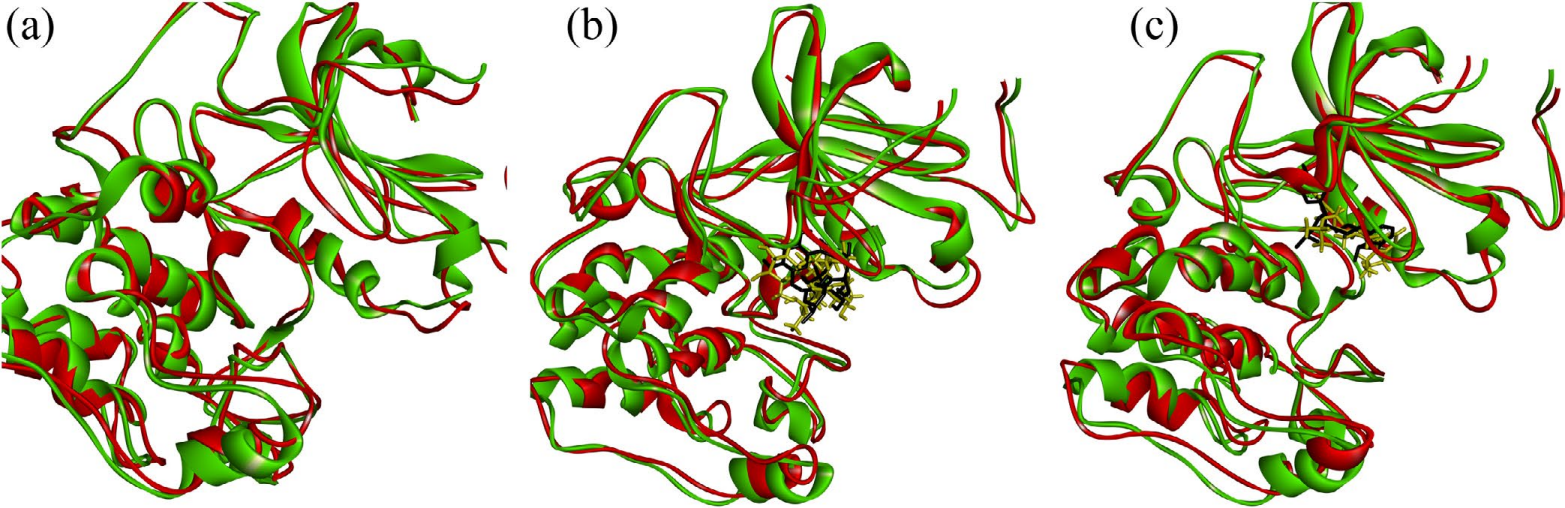
Superimposed structures of (a) Akt1 and in the presence of (b) Kaempferol 3‐rutinoside‐4′‐glucoside and (c) Ipatasertib after 110 ns computer simulations. Green and red chains demonstrate the protein chain before and after MD analyses. Black and yellow colors represent ligands before and after MD simulations, respectively. Leu156, Gly157, Gly162, Val164, Ala177, Met227, Ala230, Glu234, Glu278, Asn279, and Met281 were identified as the main residues within the Akt1 active site. Akt1, RAC‐alpha serine/threonine‐protein kinase; MD, molecular dynamics.

Figure 4 presents the structural overlay of Akt1 in three states: unbound, complexed with Kaempferol 3‐rutinoside‐4′‐glucoside and bound to Ipatasertib, illustrating the conformational changes that occurred during the 110 ns MD simulations by comparing pre‐and post‐simulation structures.

### 3.4 Interaction Modes

Following the docking simulations, a post‐docking analysis was conducted to elucidate the interactions between the top‐ranked flavonoids and critical residues within the Akt1 ATP‐binding site. The results were then contrasted with those observed for the control inhibitor. Interestingly, the analyses revealed that flavonoids primarily engaged in hydrogen bonding, while hydrophobic interactions played a lesser role. Notably, none of the flavonoids formed electrostatic bonds with the enzyme. Conversely, the control inhibitor, Ipatasertib, predominantly relied on hydrophobic interactions for binding to Akt1.

**FIGURE 5:**
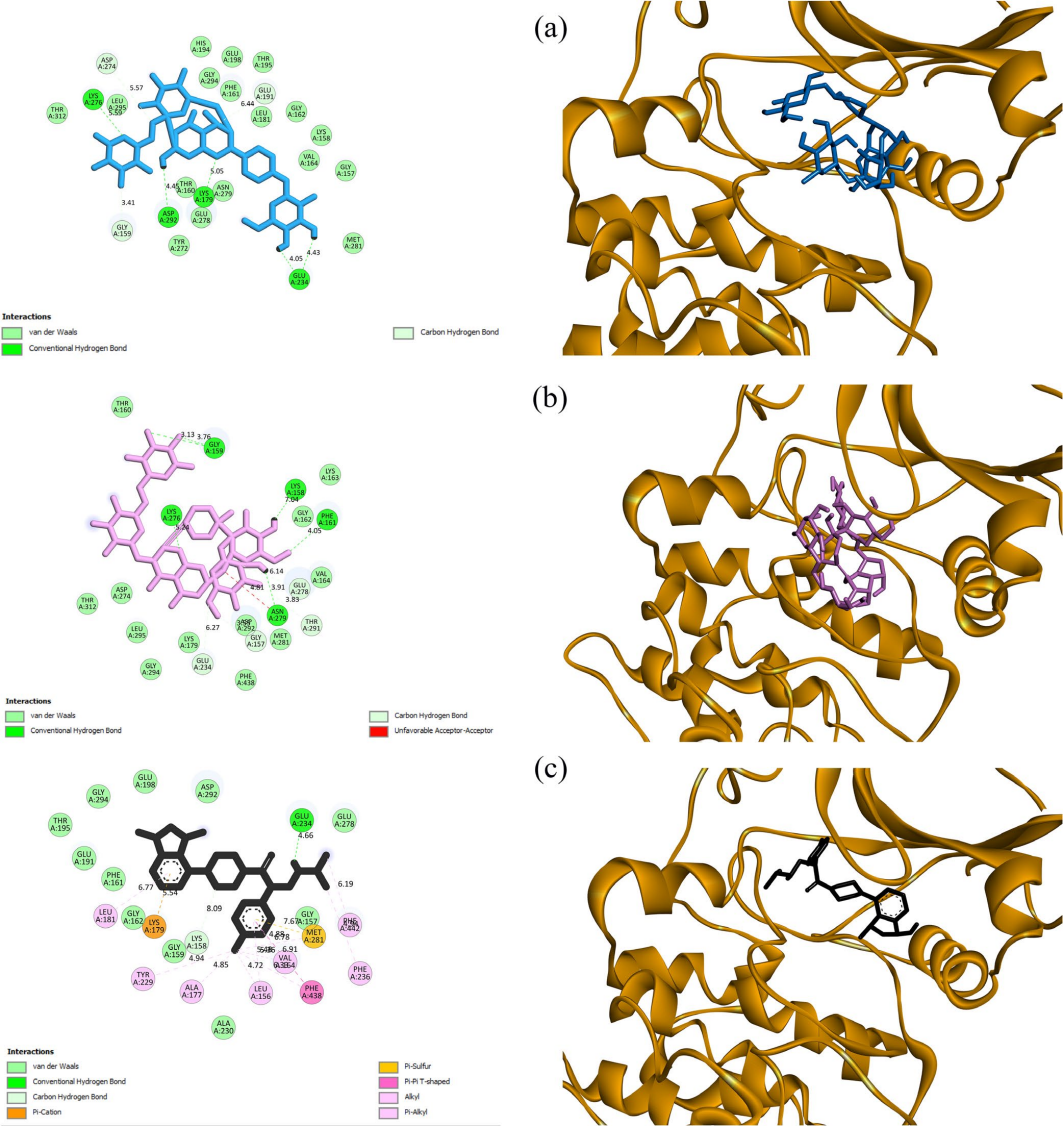
Two and three‐dimensional views of (a) Kaempferol 3‐rutinoside‐4′‐glucoside (b) Kaempferol 3‐rutinoside‐7‐glucoside, and (c) Ipatasertib within the Akt1 catalytic domain. These views were obtained from the AutoDock 4.0 software before molecular dynamics simulations. Akt1, RAC‐alpha serine/threonine‐protein kinase.

Additionally, Ipatasertib established a single electrostatic interaction with the enzyme before the MD simulation. After 100‐ns computational simulations, molecular analysis revealed that kaempferol 3‐rutinoside‐4′‐glucoside formed enhanced hydrogen bonding networks with residues in the Akt1 binding pocket and established dual hydrophobic interactions (Table 6). For a visual representation, Figure 5 depicts both two‐dimensional and three‐dimensional illustrations of Kaempferol 3‐rutinoside‐4′‐glucoside Kaempferol 3‐rutinoside‐7‐sophoroside, and Ipatasertib positioned within the active site of the receptor.

**TABLE 6:** Binding modes between the Akt1 active site, the top‐ranked flavonoids, and Ipatasertib.

Ligand name	Hydrogen bond (distance Å)	Hydrophobic interaction (distance Å)	Electrostatic interaction (distance Å)
Kaempferol 3‐rutinoside‐4′‐glucoside (before MD)	Glu234 (4.05, 4.43); Asp292 (4.45); Gly159 (3.41)	NA	NA
Kaempferol 3‐rutinoside‐4′‐glucoside (after MD)	Glu278 (3.89); Asp274 (4.65); Asp292 (5); Glu198 (4.51); Gly162 (3.49); Gly157 (3.85); Leu156 (4.02); Glu234 (4.45)	Lys276 (5.11); Val164 (5.88)	NA
Kaempferol 3‐rutinoside‐7‐sophoroside	Gly159 (3.76, 3.13); Phe161 (4.05); Gly157 (3.55); Asn279 (3.91)	Lys276 (5.19)	NA
Sophoraflavanone G	Phe161 (3.94); Asp292 (3.96); Lys276 (4.71); Asn279 (3.94)	Val164 (5.71); Lys179 (4.50); Leu181 (5.72)	NA
Amentoflavone	Gly157 (3.79); Asp292 (4.2, 4.06); Glu191 (4.63); Asn279 (3.7, 3.98)	Lys179 (5.76)	NA
Orientin	Glu198 (4.45); Gly294 (3.3); Thr160 (4.2); Gly159 (3.70); Gly162 (3.42)	Leu295 (5.16)	NA
Isoquercitrin	Lys179 (4.94); Glu278 (4.72); Asn279 (3.96); Asp274 (4.95, 4.09); Asp292 (4.45, 3.65); Lys276 (4.68)	NA	NA
Quercetin‐3‐rhamnoside	Glu234 (4.79, 4.37, 4.57); Lys179 (4.74); Gly162 (2.54); Gly157 (3.89)	NA	NA
Nicotiflorin	Gly162 (3.41); Glu278 (3.80, 3.84); Glu234 (4.94, 3.73); Asp439 (3.66)	Val164 (5.77); Phe438 (4.65); Phe442 (5.3)	NA
Rutin	Asp439 (4.42); Glu278 (4.48); Phe161 (4.79); Gly162 (3.44)	Phe442 (5.98)	NA
Ipatasertib (Before MD)	Glu234 (4.66)	Leu181 (6.77); Lys179 (5.54); Tyr229 (4.94); Ala177 (4.85); Leu156 (4.72, 5.43); Phe438 (6.33, 6.91); Val164 (5.36, 4.88); Met281 (6.78); Phe236 (4.94); Phe442 (6.19)	Lys179 (5.54)
Ipatasertib (After MD)	Glu198 (4.73); Gly294 (3.71); Thr195 (3.77)	Leu181 (5.83); Val164 (4.53, 6.46); Ala177 (4.73, 7.12); Tyr229 (4.11); Leu156 (5.28, 5.36); Phe442 (5.17)	Lys179 (5.46); Asp292 (6.1); Glu191 (8.21); Glu198 (7.92)

Abbreviations: Akt1, RAC‐alpha serine/threonine‐protein kinase; NA, not available.

### 3.5 ADMET Evaluation

The ADME properties revealed moderate blood–brain barrier penetration (0.027), acceptable water solubility, and cell permeability for Kaempferol 3‐rutinoside‐4′‐glucoside and Kaempferol 3‐rutinoside‐7‐sophoroside. Both compounds inhibited cytochrome P450 enzymes (2C19, 2C9, and 3A4), indicating potential metabolic interactions. Kaempferol 3‐rutinoside‐4′‐glucoside showed high human intestinal absorption (59.67%) with moderate plasma protein binding (25.87%), compared to 11.12% for Kaempferol 3‐rutinoside‐7‐sophoroside.

Toxicity assessments were promising: both compounds were non‐mutagenic in Ames tests, showed negative carcinogenicity in mouse and rat models, and demonstrated low acute toxicity in algae and fish studies. The inhibition of the in vitro hERG (the human Ether‐à‐go‐Related Gene) remained ambiguous and required further investigation.

The two compounds exhibited distinct ADMET parameter profiles, underscoring the significance of individual glycoside characterization for potential therapeutic applications (Table 7).

**TABLE 7:** Predicted ADMET properties of Kaempferol 3‐rutinoside‐4′‐glucoside and Kaempferol 3‐rutinoside‐7‐sophoroside.

Title	Kaempferol 3‐rutinoside‐4′‐glucoside	Kaempferol 3‐rutinoside‐7‐sophoroside
In vivo blood–brain barrier penetration (C.brain/C.blood)	0.0274612	0.0271421
Water solubility in buffer system (SK atomic types, mg/L)	4.34535	2.76156
In vitro Caco‐2 cell permeability (nm/s)	6.05627	6.8914
In vitro Cytochrome P450 2C19 inhibition	Inhibitor	Inhibitor
CYP_2C9_inhibition	Inhibitor	Inhibitor
CYP_2D6_inhibition	Non	Non
CYP_2D6_substrate	Non	Non
CYP_3A4_inhibition	Inhibitor	Inhibitor
CYP_3A4_substrate	Weakly	Weakly
Human intestinal absorption (HIA, %)	0.596744	0
In vitro Mandin Darby Canine Kidney cell permeability (nm/s)	0.0613514	0.0252411*
In vitro P‐glycoprotein inhibition	Non	Non
In vitro plasma protein binding (%)	25.866262	11.123198
Water solubility in pure water (SK atomic types, mg/L)	169.369	316.239
In vitro skin permeability (logKp, cm/h)	−4.42759	−3.8732
Calculated logD by SK atomic types in pH 7.4	−3.01962	−4.76961
Calculated logP by SK atomic types	−3.01962	−4.76961
SK logS in buffer system (SK atomic types, S: mol/L)	−5.24088	−5.52207
SK logS in pure water (SK atomic types, S: mol/L)	−3.65007	−3.46321
Acute algae toxicity	0.00436402	0.00145005
Ames test	Non‐mutagen	Non‐mutagen
Carcinogenicity (Mouse)	Negative	Negative
Carcinogenicity (Rat)	Negative	Negative
Acute daphnia toxicity	7.67916	41.4926
In vitro hERG inhibition	Ambiguous	Ambiguous
Acute fish toxicity (medaka)	121.175	3640.66
Acute fish toxicity (minnow)	94.2377	1344.84
In vitro Ames test result in TA100 strain (Metabolic activation by rat liver homogenate: Ames TA100 (+S9))	Negative	Negative
In vitro Ames test result in TA100 strain (No metabolic activation: Ames TA100 (‐S9))	Negative	Negative
In vitro Ames test result in TA1535 strain (Metabolic activation by rat liver homogenate: Ames TA1535 (+S9))	Negative	Negative
In vitro Ames test result in TA1535 strain (No metabolic activation: Ames TA1535 (‐S9))	Negative	Negative

Abbreviation: ADMET, adsorption, distribution, metabolism, excretion, and toxicity.

### 3.6 DFT Calculation

**FIGURE 6:**
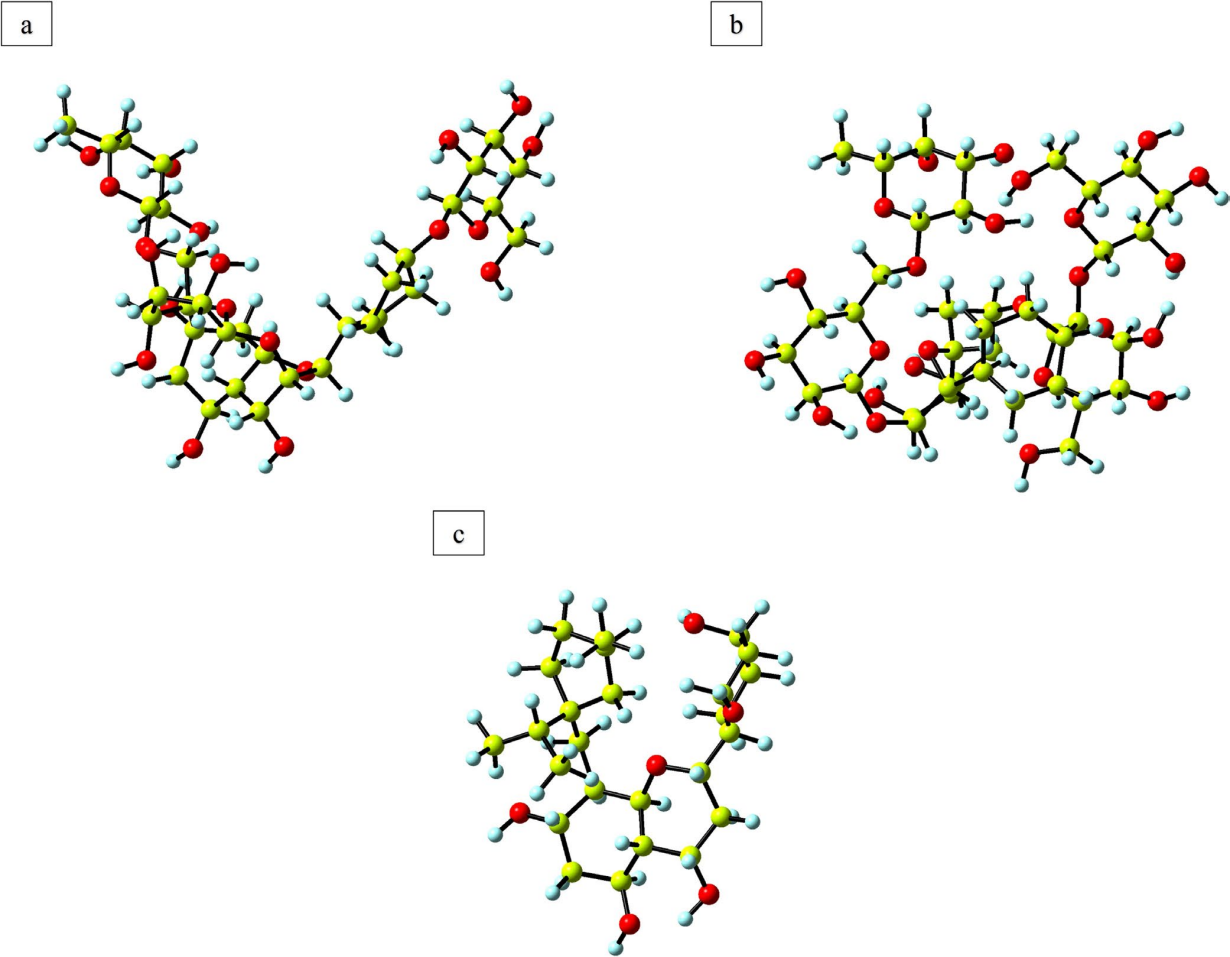
Optimized geometry of Kaempferol 3‐rutinoside‐4′‐glucoside (a), Kaempferol 3‐rutinoside‐7‐sophoroside (b), and Sophoraflavanone G (c) under the DFT method.

Based on the docking analysis, it was determined that Kaempferol 3‐rutinoside‐4′‐glucoside, Kaempferol 3‐rutinoside‐7‐sophoroside, and Sophoraflavanone G exhibited the most pronounced biological activity among the compounds tested. As a result, the DFT calculations were concentrated solely on these three compounds. The geometric optimization of these compounds was performed using the DFT/B3LYP method along with the 6‐311g (d,p) basis set. Confirming no imaginary frequencies in all optimized structures indicates that the geometries correspond to genuine local minima. The structural analysis of Kaempferol 3‐rutinoside‐4′‐glucoside, Kaempferol 3‐rutinoside‐7‐sophoroside, and Sophoraflavanone G provides valuable insights into their electronic properties. The electronic energy values observed in their ground state were −2793.94 Hartree for Kaempferol 3‐rutinoside‐7‐sophoroside, −3385.95 Hartree for Kaempferol 3‐rutinoside‐4′‐glucoside, and −1422.54 Hartree for Sophoraflavanone G. Furthermore, the calculated dipole moment values for these compounds were found to be 7.01, 12.89, and 3.35 Debye, respectively. The optimized geometries of the related compounds are illustrated in Figure 6.

The MEP map visualizes the distribution of electrostatic potential across a molecule, aiding in identifying potential binding sites. This graphical illustration offers insights into the molecular shape, size, and electrostatic properties through a distinctive color‐coding scheme. Such visualization is crucial for comprehending the relationship between molecular structure and its chemical and physical characteristics [50]. The color‐coded MEP map effectively differentiates regions that are electron‐rich from those that are electron‐deficient within the molecule. Areas characterized by low electrostatic potential, typically depicted in blue, correspond to potential donor atoms and are conducive to interactions with electron‐deficient species.

In contrast, regions shown in red signify electron‐rich zones with negative electrostatic potential, while green areas denote neutral regions with zero potential [51]. These distinct color regions are instrumental in predicting molecular reactivity. The red zones, abundant in electron density, are particularly suitable for electrophilic interactions, whereas the blue areas, associated with lower electron density, favor nucleophilic reactions [52]. This comprehensive visualization contributes to a deeper understanding of molecular behavior in diverse chemical environments.

**FIGURE 7:**
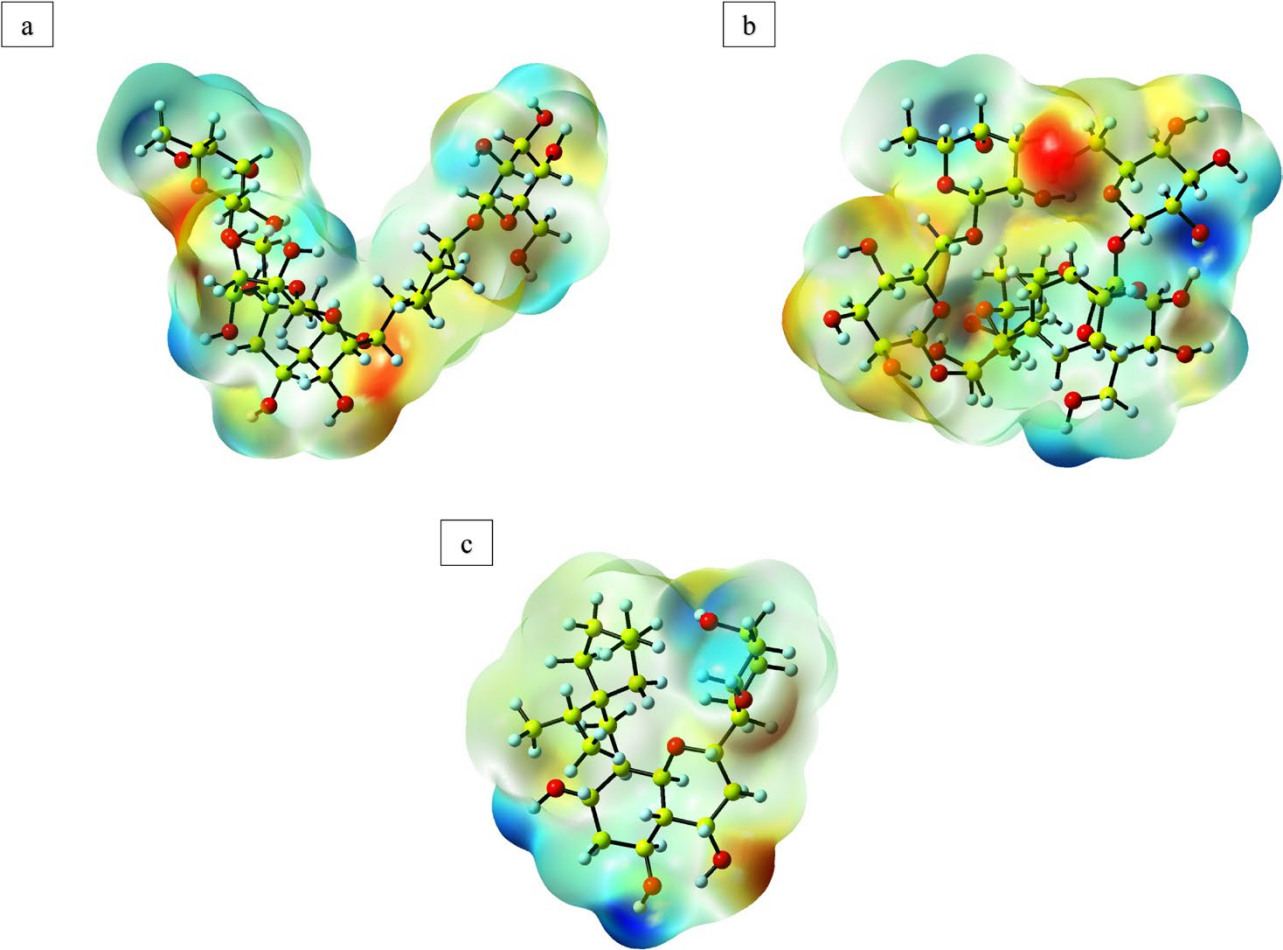
The MEP map of Kaempferol 3‐rutinoside‐4′‐glucoside (a), Kaempferol 3‐rutinoside‐7–sophoroside (b), and Sophoraflavanone G (c) under the DFT method.

The MEP maps for Kaempferol 3‐rutinoside‐7‐sophoroside, Kaempferol 3‐rutinoside‐4′‐glucoside and Sophoraflavanone G were calculated using the DFT/B3LYP/6‐311g (d,p) method, with the results illustrated in Figure 7. The MEP visualization of these compounds reveals a notable concentration of blue coloration surrounding the hydrogen atom attached to the oxygens, indicating a strong propensity for nucleophilic interactions in this region. In these maps, the oxygen atoms are highlighted in red, signifying highly favorable areas for electrophilic interactions.

**FIGURE 8:**
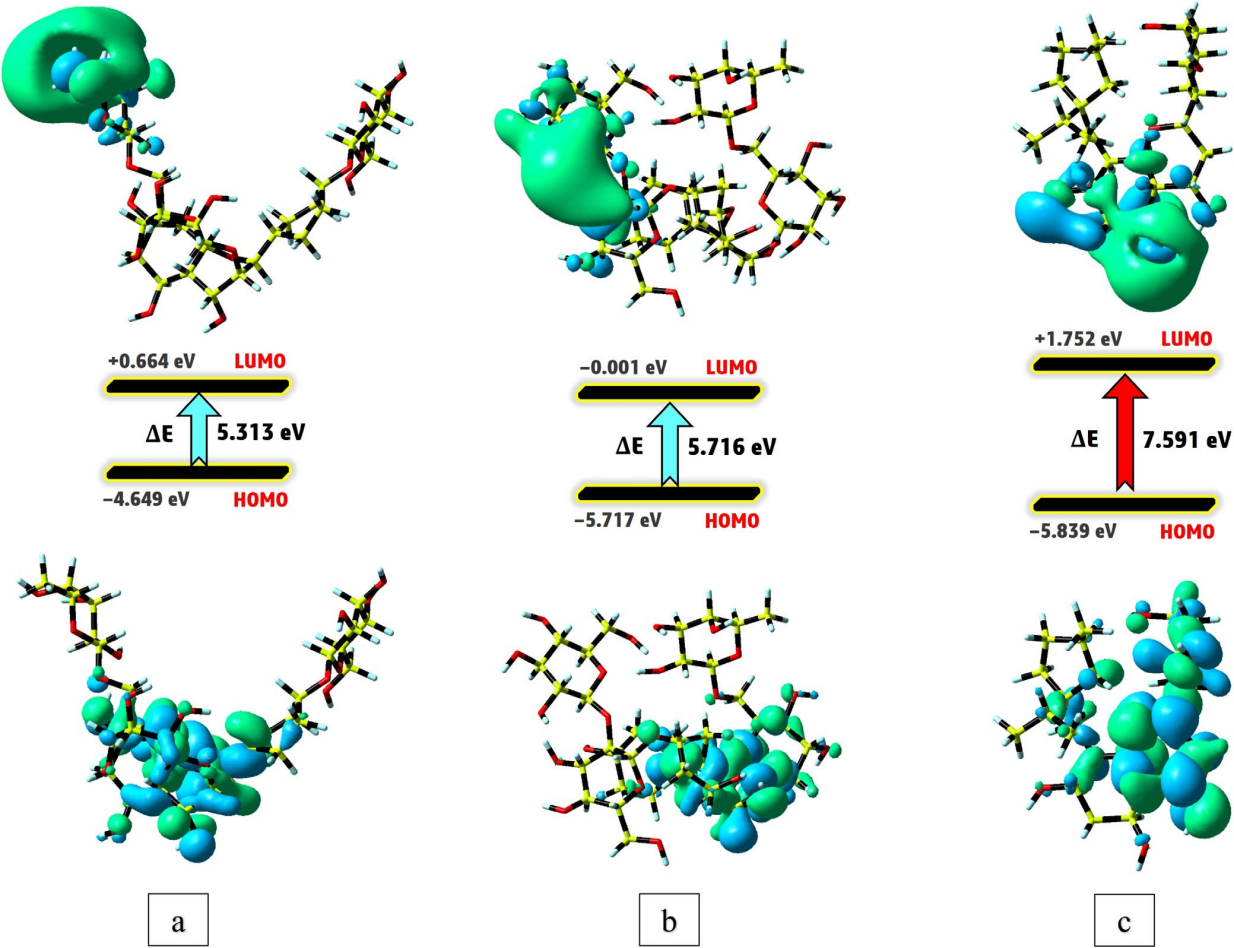
The FMOs distribution of Kaempferol 3‐rutinoside‐4′‐glucoside (a), Kaempferol 3‐rutinoside‐7‐sophoroside (b), and Sophoraflavanone G (c) under the DFT method.

The highest occupied molecular orbital (HOMO) and the lowest unoccupied molecular orbital (LUMO) are essential components of frontier molecular orbitals (FMO), significantly influencing a molecule's electronic behavior. These orbitals serve as a foundational basis for deriving insights into chemical reactivity, biological activity, and the stability of compounds [53]. Figure 8 presents the FMO contours for Kaempferol 3‐rutinoside‐4′‐glucoside Kaempferol 3‐rutinoside‐7‐sophoroside, and Sophoraflavanone G. The energy gaps calculated for Kaempferol 3‐rutinoside‐4′‐glucoside Kaempferol 3‐rutinoside‐7‐sophoroside, and Sophoraflavanone G are 5.313, 5.716, and 7.591 eV, respectively. Among the compounds examined, Kaempferol 3‐rutinoside‐4′‐glucoside is noted to exhibit a narrower energy gap compared to Kaempferol 3‐rutinoside‐7‐sophoroside and Sophoraflavanone G. This finding suggests a higher degree of chemical reactivity and potential biological activity for Kaempferol 3‐rutinoside‐4′‐glucoside albeit with diminished stability when contrasted with the other compounds. The observed trend in biological activity correlates well with the energy gap derived from docking simulations.

### 3.7 Kaempferol 3‐Rutinoside‐4′‐Glucoside Decreased the Viability of MCF‐7 Cells

**FIGURE 9:**
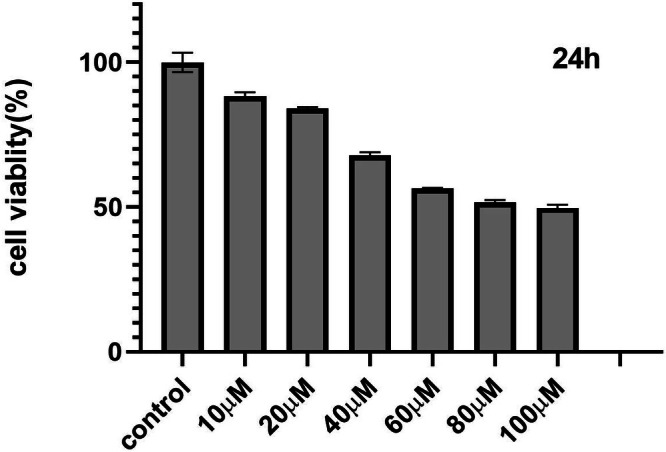
Cell viability following Kaempferol 3‐rutinoside‐4′‐glucoside treatment. MCF‐7 cells were incubated with various concentrations of Kaempferol 3‐rutinoside‐4′‐glucoside for 24 h, and viability was determined by MTT assay.

The cytotoxic effect of Kaempferol 3‐rutinoside‐4′‐glucoside (Sigma‐Aldrich; CAS No. 89439–58‐7) on MCF‐7 cells was assessed via MTT assay. MCF‐7 cells were exposed to a concentration range of Kaempferol 3‐rutinoside‐4′‐glucoside (0, 10, 20, 40, 60, 80, and 100 μM) for 24 h. As illustrated in Figure 9, a dose‐dependent reduction in the percentage of viable cells was observed in treated cells compared to untreated cells. Based on the MTT assay findings, the 50% inhibitory concentration (IC50) for Kaempferol 3‐rutinoside‐4′‐glucoside was determined to be 88.8 μM.

### 3.8 Kaempferol 3‐Rutinoside‐4′‐Glucoside Increased FOXO3 Expression in MCF‐7 Cells

**FIGURE 10:**
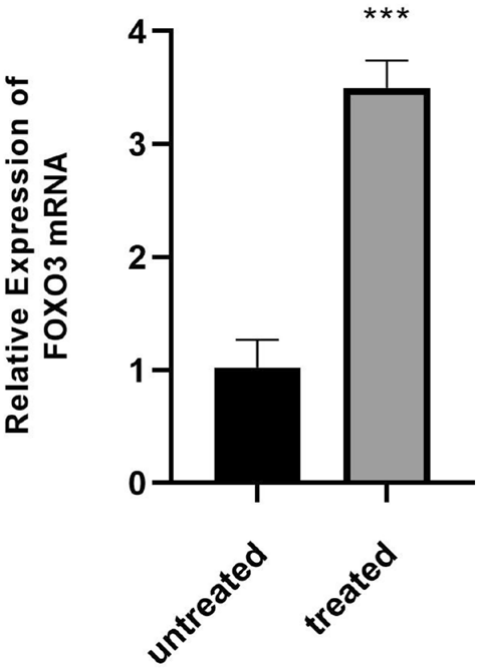
Effect of Kaempferol 3‐rutinoside‐4′‐glucoside on apoptosis‐related gene expression. RT‐qPCR analysis shows the relative FOXO3 expression (normalized to GAPDH) in MCF‐7 cells after 24 h of treatment with Kaempferol 3‐rutinoside‐4′‐glucoside compared to untreated cells. ***p‐value < 0.001 versus control group. qRT‐PCR, quantitative reverse transcription PCR.

The mRNA expression of FOXO3 was examined in MCF‐7 cells after a 24‐h exposure to 100 μM Kaempferol 3‐rutinoside‐4′‐glucoside As presented in Figure 10, treating the cells with Kaempferol 3‐rutinoside‐4′‐glucoside led to a significant upregulation of FOXO3 relative mRNA expression when normalized to GAPDH in MCF‐7 cells (*p* < 0.001).

## 4 Discussion

Akt1 plays a crucial role in the PI3K/Akt/mTOR signaling pathway, which is often dysregulated in numerous human cancers [54]. Its hyperactivation promotes proliferation, metabolism, survival, and tumorigenesis [55] and contributes to drug resistance [56], making it a promising target for anticancer drugs [55]. High‐resolution crystallographic structures of Akt1, especially those co‐crystallized with ATP‐competitive inhibitors, are readily available in the RCSB database [57]. This wealth of structural information, including details of the ATP‐binding pocket, is vital for accurate molecular docking and dynamics simulations. Many current and experimental Akt inhibitors, such as Ipatasertib (used as a control in this study), are designed to target the Akt1 ATP‐binding site due to its central role. Therefore, this study aimed to identify novel natural compounds that can specifically interfere with this crucial oncogenic signaling node by targeting Akt1, aligning with current strategies for developing more precise cancer therapeutics.

Emerging evidence has established the diverse therapeutic potential of flavonoids in cancer treatment through their interactions with multiple molecular targets within the PI3K/Akt/mTOR pathway. Zhang et al. [58] demonstrated that flavonoids effectively modulate the PI3K/Akt/mTOR pathway, inducing cell cycle arrest and apoptosis in breast cancer cells, suggesting potential analogous mechanisms for our identified compounds. The exceptional binding affinities observed in the present study for Kaempferol 3‐rutinoside‐4′‐glucoside and Kaempferol 3‐rutinoside‐7‐sophoroside gain particular significance when considered in conjunction with Petel et al.'s [59] successful experimental validation of computationally identified flavonoids as kinase inhibitors. Additionally, a comprehensive review by Mazurakova et al. [60] emphasized the critical importance of targeting this pathway in therapy‐resistant cancers, indicating that our identified flavonoids may offer promising alternatives for addressing drug resistance. This systematic computational analysis of 61 flavonoids targeting the Akt1 ATP‐binding site has yielded novel insights into the remarkable binding capabilities of Kaempferol glycosides. Notably, Kaempferol 3‐rutinoside‐4′‐glucoside and Kaempferol 3‐rutinoside‐7‐sophoroside exhibited substantially higher binding affinities compared to Ipatasertib, presenting innovative therapeutic candidates for the treatment of Akt1‐driven cancers.

Kaempferol 3‐rutinoside‐4′‐glucoside and Kaempferol 3‐rutinoside‐7‐sophoroside exhibited magnificent binding affinity to the Akt1 ATP binding cleft, with Ki values of 106.03 and 640.24 aM, respectively. The Δ*G*_binding_ values for these flavonoid‐Akt1 complexes were favorable, at −21.79 and −20.73 kcal/mol, respectively, suggesting strong binding interactions between these compounds and Akt1. The analysis also identified Sophoraflavanone G, Amentoflavone, Orientin, Isoquercitrin, Quercetin‐3‐rhamnoside, Nicotiflorin, and Rutin as potential Akt1 inhibitors. Interestingly, all these compounds displayed picomolar Ki values, indicating their ability to restrict Akt1 activity. For comparison, the reference drug Ipatasertib exhibited the Δ*G*_binding_ value of −9.98 kcal/mol. This suggests that the top‐ranked flavonoid inhibitors in this study possess a greater binding affinity for Akt1 than the established inhibitor.

The validity of the present docking results is supported by experimental evidence demonstrating the biological relevance of Akt inhibition in cancer cell lines. Our computational findings, which identified Kaempferol 3‐rutinoside‐4′‐glucosideand Kaempferol 3‐rutinoside‐7‐sophoroside as potent Akt1 inhibitors with binding affinities significantly superior to Ipatasertib (Δ*G*_binding_ = −21.79 and −20.73 kcal/mol vs. −9.98 kcal/mol for Ipatasertib), align with established experimental protocols for Akt inhibitor validation. Buckingham et al. [61] demonstrated that Ipatasertib, our reference compound, effectively inhibited cell proliferation in uterine serous carcinoma cell lines with IC50 values of 6.62 μM for PTEN wild‐type ARK1 cells and 2.05 μM for PTEN null SPEC‐2 cells, confirming its biological activity against the Akt target. The substantial improvement in predicted binding affinity observed with our top‐ranked flavonoid compounds (Ki values in the attomolar range compared to Ipatasertib's nanomolar Ki of 48.29 nM) suggests these natural compounds may possess enhanced therapeutic potential. Furthermore, our in vitro validation studies using the MTT assay and FOXO3 gene expression analysis provided experimental corroboration of the computational predictions, reinforcing the reliability of our docking methodology and the biological significance of the identified Akt1‐flavonoid interactions.

### 4.1 Plant Sources

Kaempferol glycosides exhibit a widespread distribution across diverse plant taxa. Each plant species contributes unique derivatives with distinct biological activities. Sources like horseradish ( *Armoracia rusticana* ), silver linden ( *Tilia petiolaris* DC.), *Michelia champaca* L., *Clinopodium* species, and papaya ( *Carica papaya* ) leaves exemplify the diversity and therapeutic potential of these compounds. Substantial in vitro studies support their antioxidant, anti‐inflammatory, neuroprotective, and anticancer properties [62, 63, 64, 65, 66].

Sophoraflavanone G, a prenylated flavonoid primarily isolated from *Sophora flavescens* , demonstrates a broad spectrum of biological activities. Huang et al. [67] revealed that Sophoraflavanone G induces apoptosis in triple‐negative breast cancer cells by promoting caspase activation and mitochondrial cytochrome c release. This suggests potential anticancer properties through the inhibition of the MAPK pathway.

Orientin, a flavone C‐glycoside derived from luteolin, has been synthesized using glycosyltransferase enzymes, as described by Liu et al. [68]. This example highlights the ability to generate structurally diverse flavonoids through enzymatic processes. In another study, quercetin‐3‐rhamnoside was identified among other flavonoids isolated from *Sedum japonicum* subsp. [69].

Amentoflavone is found in various plant families, including *Selaginellaceae*, *Cupressaceae*, *Euphorbiaceae*, *Podocarpaceae*, and *Calophyllaceae* [70]. This widespread occurrence suggests potential applications across diverse plant species.

Plants containing Rutin, a flavonoid glycoside composed of Quercetin and Rutinose, can be precursors for Isoquercitrin production through biochemical processes such as the Maillard reaction [71]. Rutin's ubiquitous presence in over 70 plant species underscores its therapeutic potential. Key sources include Buckwheat seeds, grapefruit, cherries, apricots, grapes, onions, plums, and oranges [72, 73].

Multiple plant sources have been identified for Nicotiflorin, including *Withania adpressa* [74], *Fraxinus hupehensis* [75], *Michelia champaca* [64], *Alternanthera brasiliana* , and wild Asparagus species [76, 77]. This diversity of sources expands the potential for obtaining this compound.

### 4.2 PI3K/Akt/mTOR Signaling Pathway Modulators

Several studies have explored the effects of various flavonoids on the PI3K/Akt/mTOR signaling pathway, a critical regulator of cell growth, survival, and proliferation. Kaempferol derivatives from *Tetrastigma hemsleyanum* roots were shown by Zhai et al. [78] to inhibit colorectal cancer cell growth. This effect was linked to the downregulation of the PI3K/Akt/mTOR pathway, confirmed by both functional assays and molecular docking studies.

Sophoraflavanone G treatment also targeted the PI3K/Akt/mTOR pathway in triple‐negative breast cancer cells, as reported by Cheng et al. [79]. Their work demonstrated that Sophoraflavanone G inhibited cancer cell growth and metastasis by downregulating EGFR expression, which in turn suppressed the PI3K/Akt pathway. This decreased the phosphorylation of Akt and downstream targets like mTOR, ultimately leading to increased oxidative stress and apoptosis.

Orientin, a flavone C‐glycoside, exhibited cardioprotective effects against hypoxia‐reoxygenation injury, according to Liu et al. [80]. Their research suggests that Orientin promotes autophagy, a cellular process regulated by PI3K/Akt/mTOR signaling. Orientin activated AMPK and Akt while downregulating mTOR phosphorylation, indicating its inhibitory effect on this pathway. This study further confirmed the role of PI3K/Akt/mTOR signaling by showing that autophagy inhibitors like wortmannin (a PI3K inhibitor) could diminish the protective effects of Orientin.

Amentoflavone's protective effects against gastric ulceration were attributed, in part, to the modulation of the AMPK/mTOR pathway, as demonstrated by Balaha et al. [81]. Their experiment involved administering different doses of Amentoflavone to rats before inducing gastric ulcers. Amentoflavone displayed a dose‐dependent protective effect, reducing ulcer formation and severity.

The anticancer properties of polyphenols found in *Ilex latifolia Thunb*., including Rutin, were investigated by Chen et al. [82]. Their study revealed that these polyphenols inhibited human lung cancer cells by regulating the PI3K/Akt signaling pathway. HPLC analysis identified Rutin as a significant component in their extract. The study suggests that these polyphenols (including Rutin) suppress key PI3K/Akt pathway elements, leading to decreased proliferation and increased apoptosis in cancer cells.

### 4.3 Interaction Modes

A comparative analysis of binding interactions revealed significant differences between flavonoids and Ipatasertib within the Akt1 active site. These disparities likely underlie the enhanced binding affinities observed for certain flavonoids, as evidenced by the following key findings:

#### 4.3.1 Interaction Types

The molecular interaction profiles of flavonoids and Ipatasertib revealed distinct binding characteristics. Flavonoids predominantly form hydrogen bonds, suggesting particular molecular interactions. Notably, Kaempferol 3‐rutinoside‐4′‐glucoside demonstrated a remarkable increase in hydrogen bond formation from four before MD simulation to eight afterward, engaging residues including Glu234, Asp292, and Gly159. In contrast, Ipatasertib exhibited fewer hydrogen bonds (one before MD, three after) and primarily relied on hydrophobic interactions.

Hydrophobic interaction analysis showed Ipatasertib formed 13 interactions before MD with residues like Leu181, Lys179, and Phe438, reducing to nine after simulation. Flavonoids generally displayed fewer hydrophobic interactions, with Sophoraflavanone G forming three, while many others formed one to two or none.

Electrostatic interactions were minimal, with Ipatasertib forming one interaction before MD (Lys179) and four after, while flavonoids showed no electrostatic interactions.

#### 4.3.2 Binding Site Interactions

Flavonoids, exemplified by Kaempferol 3‐rutinoside‐4′‐glucoside established multiple hydrogen bonds with key residues including Glu278, Asp274, Asp292, Glu198, Gly162, and Gly157. Conversely, Ipatasertib exhibited extensive hydrophobic interactions with residues such as Leu181, Val164, Ala177, and Phe442.

#### 4.3.3 Changes After MD Simulation

Post‐MD simulation, Kaempferol 3‐rutinoside‐4′‐glucoside enhanced its binding profile by increasing hydrogen bonds from four to eight and gaining two hydrophobic interactions. Ipatasertib similarly showed increased hydrogen and electrostatic interactions after molecular dynamics simulation.

Notwithstanding the significance of these computational findings, experimental confirmation of the inhibitory effects of these compounds is essential. This necessitates rigorous in vitro and subsequent clinical trial investigations.

### 4.4 Structure–Activity Relationship (SAR)

To understand the potential of herbal flavonoids as Akt1 inhibitors, their binding energies with the Akt1 catalytic site were studied. This analysis revealed the following key observations:

#### 4.4.1 Glycosylation Pattern

The most potent compounds (Kaempferol 3‐rutinoside‐4′‐glucoside and Kaempferol 3‐rutinoside‐7‐sophoroside) contain multiple glycosidic modifications. Kaempferol 3‐rutinoside‐4′‐glucoside (Δ*G*_binding_ = −21.79 kcal/mol) has glycosylation at rings C and B. Besides, Kaempferol 3‐rutinoside‐7‐sophoroside (Δ*G*_binding_ = −20.73 kcal/mol) has glycosylation at ring C and ring A, suggesting that dual glycosylation significantly enhances binding affinity.

#### 4.4.2 Position of Glycosylation

Compounds with glycosylation at ring C generally show good binding affinity (as seen in Isoquercitrin, Quercetin‐3‐rhamnoside, Nicotiflorin, and Rutin). Combining ring C glycosylation with additional sugar moieties at positions C4 or C7 leads to optimal binding.

#### 4.4.3 Ring B Modifications

Hydroxyl groups on ring B appear beneficial for binding affinity between flavonoids and Akt1. Modifying the C4's position with glycosylation (as in Kaempferol 3‐rutinoside‐4′‐glucoside) significantly improves binding affinity.

#### 4.4.4 Ring A Features

Glycosylation at position C7 (ring A) contributes positively to binding when combined with position C3 glycosylation. Sophoraflavanone G, with its unique prenylation pattern on ring A, shows relatively good binding (Δ*G*_binding_ = −15.14 kcal/mol).

#### 4.4.5 Ring C Characteristics

The C3 position on ring C appears to be a crucial point for modification. Most of the high‐affinity compounds contain substitution at this position. Rutinoside at position C3 is a common feature among several well‐binding compounds.

This SAR analysis suggests that optimal Akt1 binding is achieved through:

1. Multiple glycosylation points.
2. Position C3 (ring C) as a primary glycosylation site.
3. Additional glycosylation at either position 4C′ (ring B) or position C7 (ring A).
4. Maintenance of the basic flavonoid scaffold with appropriate hydroxylation patterns.

### 4.5 Cell Viability and FOXO3 Gene Expression

Brunet et al. [83] found that Akt1 promotes cell survival by phosphorylating and inhibiting FOXO3. Their research indicated that Akt1 phosphorylates FOXO3, leading to its movement into the cytoplasm and subsequent degradation, which in turn reduces its ability to regulate gene expression. Conversely, when Akt1 is inhibited, FOXO3 is not phosphorylated, allowing it to enter the nucleus. This nuclear entry increases FOXO3's transcriptional activity, resulting in higher FOXO3 expression within the nucleus, where it then initiates the apoptosis pathway.

Research by Tang et al. [84] indicated that Orlistat inhibits Akt activity, significantly decreasing FOXO3 phosphorylation at Thr32 and Ser253 by 45%–50% (*p* < 0.001). This inhibition leads to FOXO3 accumulation and subsequent suppression of PD‐L1 transcription in tumor cells. Similarly, in a rat model of cyclophosphamide‐induced premature ovarian failure, Zheng et al. [85] observed that Quercetin treatment reduced the levels of phosphorylated Akt and phosphorylated FOXO3a while increasing total FOXO3a expression. Quantitatively, Quercetin improved ovarian function markers by lowering the ratio of phosphorylated Akt to total Akt and phosphorylated FOXO3 to total FOXO3.

### 4.6 A Similar Study Using Flavonoids

Our computational approach for identifying flavonoid‐based Akt1 inhibitors shares methodological similarities with recent virtual screening studies, including the work of Saha et al. [86], who employed molecular docking, MD simulations, and DFT analysis to identify flavonoids as potential therapeutics against *Naegleria fowleri* CYP51 for primary amoebic meningoencephalitis treatment. Both studies demonstrate the therapeutic versatility of flavonoids across diverse disease contexts, with Saha et al. [86] reporting binding affinities ranging from −10.9 to −10.6 kcal/mol for their top hits (beta naphthoflavone, abyssinone I, and abyssinone III), which align closely with established molecular docking benchmarks in the literature. In contrast, our study revealed exceptionally high binding affinities for Kaempferol glycosides (ΔG ≤ −20 kcal/mol). These values exceed those typically reported in flavonoid docking studies and may reflect differences in target protein characteristics, binding site properties, or docking parameters. However, a critical advantage of our investigation over purely computational studies such as that by Saha et al. [86] is the incorporation of in vitro experimental validation through cell viability assays and FOXO3 gene expression analysis in MCF‐7 cells, demonstrating that our computational predictions translated to measurable biological effects. The alignment between our docking results and in vitro outcomes strengthens confidence in the therapeutic potential of the identified Kaempferol derivatives and validates our computational methodology. These findings contribute to the growing body of evidence supporting flavonoids as promising therapeutic scaffolds, whether targeting oncological pathways as demonstrated in our study or addressing parasitic infections as shown by Saha et al. [86], highlighting the broad‐spectrum potential of these natural products in drug discovery efforts across multiple disease areas.

This study is limited by the lack of interaction probability analysis across the MD simulation; this was not feasible due to software restrictions and should be addressed in future investigations to provide deeper insights into contact stability dynamics.

## 5 Conclusion

This study represented Kaempferol 3‐rutinoside‐4′‐glucoside and Kaempferol 3‐rutinoside‐7‐sophoroside as potent Akt1 inhibitors with significantly superior predicted binding affinities compared to Ipatasertib. MD simulations further supported the stability of the Kaempferol 3‐rutinoside‐4′‐glucosideAkt1 complex. These in silico findings were corroborated by experimental in vitro results. This highlights the promising therapeutic potential of specific flavonoids as Akt1 inhibitors and warrants further in vivo validation to confirm their efficacy in cancer treatment.

## Author Contributions

A.T. and S.J. designed the study. A.E. conducted docking operations. A.T. and M.F.‐D. executed MD and DFT calculations, respectively. All authors discussed the results. A.T. and M.F.‐D. wrote the manuscript. All authors read and approved the final version of the manuscript.

## Ethics Statement

The present study has been confirmed by the Ethics Committee of Hamadan University of Medical Sciences, Hamadan, Iran (IR.UMSHA.REC.1402.231).

## Consent

The authors have nothing to report.

## Conflicts of Interest

The authors declare no conflicts of interest.

## Data Availability

The data that support the findings of this study are available from the corresponding author upon reasonable request.
